# Explainable Machine Learning-Based Overall Survival Classification in Prostate Adenocarcinoma Using Integrated Clinical and Transcriptomic Features

**DOI:** 10.3390/diagnostics16091345

**Published:** 2026-04-29

**Authors:** Hasan Anıl Kurt, Sabire Kılıçarslan, Merve Meliha Çiçekliyurt

**Affiliations:** 1 Department of Urology, Faculty of Medicine, Çanakkale Onsekiz Mart University, 17020 Çanakkale, Turkey; anilkurt@comu.edu.tr; 2 Department of Medical System Biology, Graduate School of Sciences, Çanakkale Onsekiz Mart University, 17020 Çanakkale, Turkey; sabire.kilicarslan@gmail.com; 3 Department of Medical Biology, Faculty of Medicine, Çanakkale Onsekiz Mart University, 17020 Çanakkale, Turkey

**Keywords:** prostate adenocarcinoma, machine learning, explainable AI, survival classification, Cox regression

## Abstract

**Background/Objectives**: Prostate adenocarcinoma exhibits substantial inter-patient heterogeneity, limiting the accuracy of current prognostic tools. Prostate-specific antigen-based assessment remains insufficient for reliable survival prediction. There is a clear need for integrative, data-driven approaches that leverage multi-dimensional clinical and molecular data to improve outcome stratification. This study aimed to develop and evaluate an explicable machine learning framework for predicting overall survival in prostate adenocarcinoma. **Methods**: A comprehensive machine learning pipeline was constructed using clinical and laboratory data from 494 patients in the TCGA PanCancer Atlas cohort. Following data curation, 16 clinically relevant features were selected through expert-guided filtering and feature selection techniques. Missing values were addressed using imputation strategies, and class imbalance was mitigated using SMOTE. Eight machine learning models were evaluated, including a novel hybrid ensemble model combining Gradient Boosting Machine and random forest (GBM + RF). Model performance was assessed using stratified 10-fold cross-validation and quantified via accuracy, precision, recall, F1-score, and ROC-AUC. Model interpretability was examined using LIME, and prognostic relevance was validated through Cox proportional hazards regression. **Results**: The hybrid GBM + RF model demonstrated superior performance, achieving 97% accuracy and a ROC-AUC of 0.95 under mode imputation with SMOTE balancing. Ensemble-based models consistently outperformed single classifiers, particularly in handling missing data and class imbalance. Key predictors of survival included progression-free survival, hypoxia-related scores, genomic instability markers, and immune-associated variables. Cox regression analysis confirmed the independent prognostic significance of these features, supporting the biological plausibility of the model. **Conclusions**: An explainable ensemble machine learning approach enables accurate and clinically interpretable prediction of overall survival in prostate adenocarcinoma. The proposed framework provides a robust foundation for precision urology decision-support systems and warrants validation in independent cohorts.

## 1. Introduction

Prostate cancer is a serious public health problem that physicians most frequently encounter in men, on a per diagnosis basis, than other types of cancer and one which is listed as one of the most lethal cancers worldwide [1]. Most patients have a slow variant that remains localized within the gland, but a significant portion of them have a rapidly progressing, biologically aggressive type. The tumors spread beyond their original location at an early stage and they show resistance to conventional treatment methods [2]. Prostate adenocarcinoma (PAC) serves as the main histologic subtype because approximately ninety to ninety five percent of tumors develop from glandular tissue [1,3]. Clinicians still face difficulties with predicting a patient’s overall survival duration despite advancements in screening technology and molecular research and new drug development efforts [4].

Patients who receive early diagnosis of their health condition show better treatment results compared to those who receive later medical evaluation. Prostate cancer diagnosis at its initial stage allows for surgical removal in certain situations while advanced treatment options through specific newer medications lead to substantial patient quality-of-life gains according to some studies [5,6]. The patient’s symptoms will change after the disease progresses beyond its initial stage because the body will develop serious medical problems, while the treatment becomes less effective at maintaining life expectancy according to research findings [7,8]. Medical diagnostic tools have achieved progress in sensitivity and specificity detection abilities yet they continue to present important limitations. PSA testing is not specific enough for accurate decision-making and therefore it remains unused. On the other hand, cytology testing fails to detect low-grade prostate tumors which doctors frequently find to be problematic cases according to research findings [9,10].

Researchers studying tumor behavior face major challenges recognizing tumors. Every tumor has different characteristics, which in turn make researchers’ work studying their development patterns very difficult. Medics too have a tough time because of tumors that grow in unpredictable ways presenting obstacles that make it difficult for them to be observed. The machines which we use for imaging the inner parts of our bodies, which take pictures of our insides, are not very good at finding such tumors.

The research field of machine learning (ML) today serves as an effective analytical method which enables researchers to analyze complex clinical data that contains multiple types of high-dimensional information [11,12,13]. The generalization performance of ensemble learning methods which include bagging and boosting as well as stacking and voting-based techniques achieves better results through their method of combining multiple base learners into a single predictive system. These methods generate decrease variance results that not only guard against overfitting but also boost system strength. As a result, they became highly efficient for the prediction of survival outcomes based on various types of oncology datasets.

The previous research on prostate cancer (PCa) diagnostic methods and subtype identification using machine learning (ML) techniques has laid down a foundation of evidence which is still fragmented and incomplete, as it only looks at ensemble-based methods for predicting overall survival in PAC patients. Most existing research either employs single-model architectures or evaluates simplified ensemble strategies without a systematic comparative analysis of multiple advanced boosting and bagging frameworks. The complete examination and combination of genomic instability markers and hypoxia scores together with mutation-derived features into a unified ensemble survival model remains unstudied.

The current study establishes multiple ensemble machine learning models to predict overall survival in prostate adenocarcinoma by using the TCGA PanCancer Atlas cohort for its research work. The study combines clinical data with genomic data and molecular data through an ensemble framework that has undergone thorough validation to achieve better prognostic accuracy which will serve as the foundation for future clinical decision-support system development.

The researchers in this study established model interpretability and clinical transparency as essential elements of their design process according to their research design framework. The predictive system used LIME and SHAP as built-in systems which allowed users to see which elements affected survival risk scores at two different levels of analysis. The research team created a complete preprocessing system that used multiple methods to handle missing information together with SMOTE resampling to solve problems related to class distribution. The model combines clinical and genomic data into a single analytical system which improves biological accuracy and its potential uses in precision oncology research. The key contributions of this study are outlined as follows:

- We created a new hybrid model that combines GBM and RF to forecast survival rates in prostate adenocarcinoma patients.
- The system incorporates LIME and SHAP as part of its design because the output transforms into interpretable information that doctors can understand.
- The preprocessing system we implemented uses advanced techniques to address missing values while it maintains balance between different classes through SMOTE implementation.
- The research team conducted a direct comparison between eight distinct ML algorithms using identical testing conditions to determine which algorithm performed best.

### 1.1. Motivation

The research study investigates whether the explainable hybrid ensemble machine learning system better predicts prostate adenocarcinoma overall survival than existing single and simplified ensemble machine learning systems. Existing research mainly investigates recurrence prediction while using a single prediction model and considers interpretability as a secondary process that occurs after creating models. The current research investigates overall survival through direct modeling while building explainability into the entire modeling process. The work introduces three original elements: (i) the creation of a dedicated hybrid GBM + RF ensemble which serves as a custom solution for OS classification, (ii) the systematized use of LIME and SHAP which enables both comprehensive and individual patient visibility, and (iii) the establishment of a comprehensive data analysis system that uses multiple imputation methods with SMOTE class balancing and stratified cross-validation to provide robust solutions for actual data situations. The study represents the first research to create a hybrid ensemble framework which explains its structure through combined clinical and transcriptomic data for predicting prostate adenocarcinoma overall survival.

### 1.2. Literature Review

A substantial body of research has been conducted on PAC, with a considerable portion of the published literature being exploratory in nature. In this work, we examine the historical, current, and prospective scientific studies on PAC. Guo as well as their co-workers used a prognostic model to define the immunity-linked adenocarcinoma subtypes of PAC and underlined the molecular diversity of the disease [14].

During the past few years, more researchers have become interested in using ensemble models to diagnose cancer [15]. The main variants are stacking, hard voting, soft voting and weighted voting. Stacking trains multiple base classifiers—decision trees, random forests, k-nearest neighbors plus extreme gradient boosting—then learns how to combine their outputs into a single strong predictor [16]. Soft voting usually provides better cancer classification results than rigid voting [17]. Hard and soft voting both raise diagnostic accuracy but have also proved useful in medicine, for example in classifying ischemic stroke [18].

Prior studies have increasingly applied ML to prostate cancer prognostication, and more recent work has emphasized the use of explainable AI to improve interpretability and support clinical translation. Prognostic modeling in prostate cancer is rapidly shifting from static nomograms [15,16,17] toward machine learning (ML) frameworks capable of navigating high-dimensional, multimodal data [18,19,20]. Recent benchmarking suggests that ML algorithms, particularly ensemble methods, outperform classical models in predicting overall survival (OS) across diverse clinical settings, from lymph node-positive disease to metastatic cohorts [21,22,23].

The real power of these models, however, lies in their ability to fuse clinicopathologic variables with molecular insights. By integrating TCGA-driven gene signatures, single-cell RNA-seq data, and even radiomic features from PET/CT imaging, researchers are achieving a level of prognostic resolution that clinical markers alone cannot provide [24,25,26,27]. Yet high performance often comes at the cost of transparency. To bridge this “black-box” gap, recent efforts have focused on explainable AI (XAI) and SHAP-based frameworks, ensuring that model predictions are not just accurate but interpretable for bedside decision-making [28,29].

Despite this momentum, the field still struggles with fragmented validation. Many models remain “locked” within single-institution datasets, raising valid concerns about their real-world generalizability and potential overfitting [18]. Furthermore, inconsistent reporting on data handling—such as how missing values or class imbalances are managed—continues to hinder clinical translation [18]. Our study addresses these shortcomings by developing an XAI-supported prognostic framework that merges molecular and clinical data, prioritizing both predictive rigor and transparent risk stratification.

Kilicarslan et al. (2026) applied XGBoost, random forest, and other machine learning techniques to predict overall survival in patients with castration-resistant prostate cancer, reporting that ensemble models outperformed traditional statistical approaches in terms of C-index [30]. Similarly, Emegano et al. (2025) conducted overall survival analyses in prostate adenocarcinoma patients using LightGBM, CatBoost, and random forest, demonstrating improved prognostic accuracy through ensemble learning strategies [21]. Zhang et al. (2025) developed an interpretable XGBoost-based survival prediction model for patients with bone-metastatic prostate cancer, highlighting its potential to support clinical decision-making for 1-, 3-, and 5-year survival outcomes [31]. Ahuja et al. (2024) utilized clinical datasets to implement ANN, random forest, and AdaBoost models for survival prediction and treatment recommendation in prostate cancer patients [32]. Janbain et al. focused on predicting biochemical recurrence using a Random Survival Forest model and compared its performance with conventional Cox regression [33]. Finally, Peng et al. developed prognostic machine learning models, including GBSA, RSF, and EST, to estimate overall survival in lymph node-positive prostate cancer patients, benchmarking their performance against Cox regression analyses [23,34].

Progress in predicting and modeling prostate adenocarcinoma has not yet led to wide use of ensemble methods for forecasting overall survival. Traditional statistical models plus single machine learning algorithms usually fail to show how clinical, pathological and molecular factors interact. Ensemble methods combine multiple algorithms and therefore raise both accuracy but also stability. Yet they have rarely been tested on biopsy data for survival classification in prostate adenocarcinoma. Earlier work has mostly used one algorithm or only simple ensemble schemes—thorough studies that test and compare advanced ensemble techniques are still required.

## 2. Materials and Methods

In this study, we worked with one dataset from cBioPortal for Cancer Genomics (https://www.cbioportal.org/datasets): prostate adenocarcinoma (TCGA, PanCancer Atlas) (accessed on 16 February 2026) [35] https://www.cbioportal.org/study/summary?id=prad_tcga_pan_can_atlas_2018 (accessed on 16 February 2026). These datasets included 494 samples. Since the files were originally in TSV format, we converted them to CSV format to make them easier to work with during our analysis.

The downloaded prostate adenocarcinoma (TCGA, PanCancer Atlas) datasets consist of 63 columns and 494 rows, respectively. In both datasets, variables with all values marked as “NA”, as well as those unrelated to the scope of the study such as patient identification (ID) and hospital codes were excluded from the analysis.

**Figure 1:**
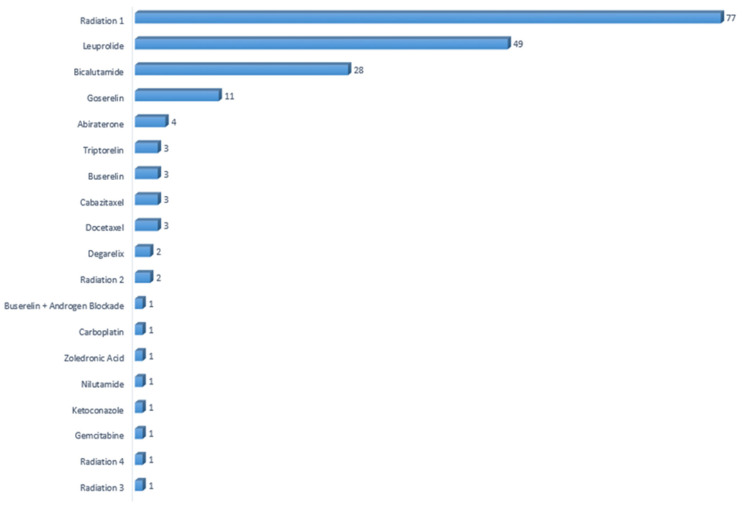
Number of patients per treatment type in prostate adenocarcinoma.

This study primarily focused on clinically and biologically relevant features that are associated with each cancer type. In the dataset, 19 distinct treatment strategies were recorded for prostate adenocarcinoma. The number of cases corresponding to each treatment method are visualized in Figure 1.

The institutional origins of the data used in this study are obvious: they derive predominantly from revered cancer research centers, mostly in the United States, and some in Canada, with several notable contributions from institutions in Europe and South America.

For this study, the prostate adenocarcinoma dataset contained contributions from 11 institutions. The two largest samples came from, in order, the University of Pittsburgh (96 samples, 19.4%), and the International Genomics Consortium (63 samples, 12.8%). Meanwhile, MD Anderson Cancer Center and Roswell Park Comprehensive Cancer Center contributed 45 (9.1%) and 43 (8.7%) samples, respectively. Other major contributors were Indivumed (Hamburg, Germany, 32 samples, 6.5%), University Medical Center Hamburg-Eppendorf (Hamburg, Germany, 24 samples, 4.9%). Procure Biobank (Montreal, QC, Canada, 27 samples, 5.5%), University of California, San Francisco, CA, USA (24 samples, 5.5%), and ABS—Lahey Clinic (Burlington, MA, USA, 24 samples, 5.5%) also made major contributions to the dataset. Overall, these figures indicate that numerous institutions were involved in the TCGA data gathering process. The number of research centers in North America, particularly in the U.S., is astonishing. On the other hand, the participation from countries like Germany, the UK, and Brazil clearly demonstrates the collaborative and global nature of cancer genomics research.

This formulation enabled unified benchmarking across heterogeneous classifiers, improved robustness under class imbalance, and facilitated seamless integration with ensemble learning and understandable artificial intelligence methods. While time-to-event survival models remain valuable, the binary framework was intentionally adopted to support understandable and clinically actionable decision-making.

The data analyzed in this study is a combination of clinical and molecular information from 494 patients, all of whom were diagnosed with prostate adenocarcinoma (PAC). The data was derived from the TCGA PanCancer Atlas and contains patients who have been classified according to the clinical and biological features of their prostate cancer. The classes of the endpoints relate to the overall survival (OS) and are in the form of binary categories: 0 (alive) and 1 (deceased). The labeling gold standard was the clinical follow-up records and the actual survival outcomes of the patients. This classification is confirmed by the clinical data in the TCGA dataset.

### 2.1. Preprocessing Process

For this study, the prostate adenocarcinoma dataset (TCGA, PanCancer Atlas) was sourced from the cBioPortal platform, initially comprising 63 columns and 494 rows. The data were cleaned in advance so that the model would give more reliable answers and would not be confused by useless entries. Columns that carried almost no information—study titles, patient IDs, AJCC codes, tumor stage labels, etc.—were dropped, as were any columns that held only blanks. After this step, the file held 16 variables that clinicians or genomicists judged to be relevant for the estimation of how long a patient would live. To streamline subsequent data input and mitigate the potential for typographical errors, each variable name was truncated into a succinct label. In the dataset, the “OSS” (Overall Survival Status) column contains 484 samples, the majority of which represent patients who are alive (0). In contrast, only 10 samples represent patients who have died (1).

The selected variables and their corresponding abbreviations are as follows:

- DA (Age at Diagnosis)
- AS (Aneuploidy Score)
- BHS (Buffa Hypoxia Score)
- LCC (Last Contact Date After Initial Diagnosis)
- DFM (Number of Months Disease-Free)
- MDSC (Disease-Specific Survival Time—in months)
- FGA (Fraction Altered in Genome)
- MSIMan (MSI MANTIS Score)
- MSISen (MSIsensor Score)
- MC (Number of Mutations)
- OVM (Overall Survival Time—in months)
- OSS (Overall Survival Status; 0 = alive and 1 = deceased)
- PFS (Progression-Free Survival—in months)
- RHS (Ragnum Hypoxia Score)
- Sex (Gender)
- TMB (Tumor Mutation Load)

In order to determine the most significant variables in the analysis, a one-way analysis of variance (ANOVA) [36] test was applied. We did feature selection in two steps. First, we used ANOVA to filter out variables that do not give us information. Then, we used Recursive Feature Elimination (RFE) to look at how variables interact with each other and to narrow down the list of features. The main goal of this test is to find out which variables are really important for predicting the survival (OS) of patients. We want to know which variables are associated with survival (OS). The patients’ overall survival was studied carefully. Some things were found to be really important for patients’ survival. These things were saved to be used for making the models. The overall survival of the patients was looked at like a yes-or-no question, to make it easy to compare the results from different machine learning methods. In this study, the overall survival of the patients was treated like a question that can only be answered in two ways, rather than trying to figure out exactly how long the patients would survive. The overall survival of the patients was the focus and it was looked at in a way that made it easy to understand the results. We found it was easier to work with the machine learning classifiers when we used methods that help us understand the results of the machine learning classifiers. The main thing we were looking at was survival and we used it to train the models in a way that made sense for this study on overall survival. We made this decision because our main goal was to make it possible to compare the machine learning classifiers, even though they are all different, and to make it easy to use them with ensemble learning and explainable AI methods. Time-to-event models like Cox regression or survival forests give us an idea of what happens over time. Binary classification is more useful when doctors need to make quick decisions. This is because binary classification is easier to understand and it helps doctors figure out who is at risk. Binary classification is very helpful in these situations. In the future we will try to use this method with time–survival modeling, which also uses time-to-event models, and we will use survival forests to make it even more useful for doctors and binary classification.

The doctors looked at information that can affect how long people live. Things like how bad the disease gets over time are connected to how long people live. To make sure the information is fair, the team only used the training data to get the information ready. This means they only picked the information, filled in the missing pieces, and made sure the data was balanced when they were using the training data to test the model. The team knows that using information from later on can make the model look better than it really is. The team tested the model’s efficacy with the data they had from the start. They wanted to verify that the model is stable and works with the clinical and molecular features they have.

As a result of the ANOVA test, it was decided to include a total of 16 variables in the advanced modeling stage. It was judged that these variables have the strongest potential to contribute to survival classification in the context of prostate cancer. These features constituted the basic analytical dataset used in the advanced stages of the study. Table 1 shows the results of the variables that had a statistically significant effect on overall survival. The statistical significance levels in these tables suggest that the 16 chosen variables are appropriate for predictive modeling.

After meticulous data cleaning, several imputation techniques were employed, such as mean, median, mode, and k-nearest neighbor (KNN) methods. [14]. The issue of class imbalance was solved by implementing the synthetic minority oversampling technique (SMOTE) [37] for oversampling and an ensemble-based approach for undersampling [38]. The performance of the models was measured rigorously with the use of standard classification metrics like accuracy, precision, recall, F1-score, and ROC-AUC [39], thus being sure that all developed models had a thorough assessment of predictive effectiveness. The model fused genomic data with clinical data to elevate accuracy. Missing values after meticulous data cleaning were filled with mean, median, mode, or k-nearest neighbor methods. Class imbalance was rectified—using oversampling through SMOTE—as was undersampling using an ensemble strategy. It created synthetic examples for the minority class (deceased patients), thus rebalancing the class distribution. After SMOTE, there were an equal number of samples from both classes: 436 samples for each class. This move lessened the influence of class imbalance and enabled the model to gain knowledge equally from both classes. It also made the level of performance evaluation fair. The performance of the models was evaluated using accuracy, precision, recall rate, F1-score, ROC-AUC, and training time, ensuring that the predictive ability of each model was fully checked. In this study, we used 10-fold cross-validation as a data splitting strategy. The method divides the data into 10 equal parts, using use one part as a validation set while the nine remaining parts serve as training data. The process continues for 10 iterations which use each part as a validation set. Cross-validation enables researchers to evaluate model performance more accurately because it employs all data points for model training and testing while also preventing data leakage and assessing the model’s ability to generalize.

### 2.2. Imputation Strategies

Dealing with missing data is one of the key steps in the preprocessing stage, since it very much influences the model’s performance and trustworthiness. In this research, four various imputation methods were applied and the prediction accuracy with each strategy was assessed. The mean imputing method takes missing values to be the mean of the variable concerned, thus providing simplicity and being efficient in computation, but on the other hand, it may introduce bias in datasets with high variance or skewed distributions [40]. The median imputation technique is, however, more robust to outliers using the median value of the variable and hence is appropriate for non-normal distributions that are often seen in real-world data [40,41]. In the case of categorical variables, mode imputation was used, which populates missing entries with the most frequent category; while being quick and easy, it can cause an over-representation of leading classes and thus requires close observation of category balance [40,41]. K-nearest neighbor (KNN) imputation [42], on the other hand, is a method of missing value estimation that considers the values of the k nearest data points (k = 5 in this study), thus maintaining structural relationships within the dataset and yielding realistic estimates, especially for continuous variables. Nonetheless, it is more computationally intensive and requires the data to be scaled and normalized properly to ensure that the distance calculations are meaningful.

### 2.3. Machine Learning Algorithms

Machine learning uses data to build models automatically. A system learns how to run a chosen algorithm and then pulls out facts that are not obvious. The system receives no directions about where to look—the model alone finds useful regularities in the data. Each pass through the data lets the model adjust its steps when new cases appear—hospitals spend less plus patients talk more easily with their doctors. When the dataset is massive, machine learning algorithms serve as practical instruments for finding and studying intricate patterns. [43,44].

Machine learning algorithms now change quickly when they serve medicine, especially when they improve a single patient’s treatment plan and when they build practical health systems. Big-data tools strengthen machine learning and give it many openings for use in health care. Doctors use it to give each patient a prescription and a treatment plan that fits that patient alone but also it tells the patient the exact day and hour to book the next visit. [45].

### 2.4. Proposed Method

In this study, a hybrid machine learning model combining the strengths of Gradient Boosting Machine (GBM) and random forest (RF) algorithms was designed to improve overall survival (OS) prediction in patients with prostate adenocarcinoma (PAC) [46]. The model was structured with the goals of achieving high accuracy, reducing variance, and maintaining clinical interpretability.

Gradient Boosting Machine (GBM)

GBM [27] sequentially produces weak learners (usually decision trees) to iteratively correct the errors of the previous model. The objective is to minimize a selected loss function L(y,F(x)) (Equation (1)).

Initial prediction:

(1) $$F_{0}\left(x\right)=arg\;\underset{\gamma }{\min}\sum_{i=1}^{n}L(y_{i},\gamma )$$

At each iteration, the negative gradient (pseudo-residual) is calculated (Equation (2)):

(2) $$r_{im}=-{\left[\frac{\partial L(y_{i},F\left(x_{i}\right))\;}{\partial F\left(x_{i}\right)}\right]}_{F=F_{m}-1}$$

The weak learner is trained on these residual values. The step size ($\gamma_{m}$) is determined as (Equation (3))

(3) $$\gamma_{m}=arg\;\underset{\gamma }{\min}\sum_{i=1}^{n}L(y_{i},F_{m-1}\left(x_{i}\right)+\gamma h_{m}\left(x_{i}\right))$$

Model update (Equation (4)):

(4) $$F_{m}\left(x\right)=F_{m-1}\left(x\right)+v.\gamma_{m}.h_{m}(x)$$

where ν is the learning rate.

Random Forest (RF)

RF is an ensemble method that averages the predictions of many decision trees built using bootstrap sampling and random feature selection [47]. Each tree T_b (x) is trained on a randomly selected subset of the data. Final prediction (Equation (5)):

(5) $$\hat{f}RF\left(x\right)=\frac{1}{B}\sum_{b=1}^{B}T_{b}(x)$$

This method:

- Reduces variance,
- Prevents overfitting,
- Provides feature importance scores for interpretability.

Hybrid GBM + RF Model

The proposed hybrid GBM + RF model integrates two powerful ensemble learning algorithms in a sequential manner. In the first stage, GBM captures complex, nonlinear, and interactive relationships between features and the target variable by producing sequential weak learners $h_{m}(x)$. GBM starts with a base prediction function $F_{0}(x)$ and, at each iteration, computes the negative gradient (pseudo-residual) r_im_ to identify the direction of steepest descent in the loss function L(y,F(x)). Each new weak learner is trained on these residuals, and the model is updated using a step size γ_m_ and learning rate ν (Equation (6)):

(6) $$F_{m}\left(x\right)=F_{m-1}\left(x\right)+v.\gamma_{m}.h_{m}(x)$$

At the end of this process, intermediate prediction scores ($\hat{y}\text{\_}GBM$) are generated for each observation. In the second stage, these scores are concatenated with the original feature matrix X to create an extended feature space $Z\;=\;[X,\hat{y}\text{\_}GBM]$. The RF model is then trained on this extended space using bootstrap sampling and random feature selection, building B independent decision trees (T_b) (x). The final hybrid model prediction is (Equation (7)):

(7) $$\hat{y}Hybrid\left(x\right)=\frac{1}{B}\sum_{b=1}^{B}T_{b}([x,\hat{y}GBM])$$

**Figure 2:**
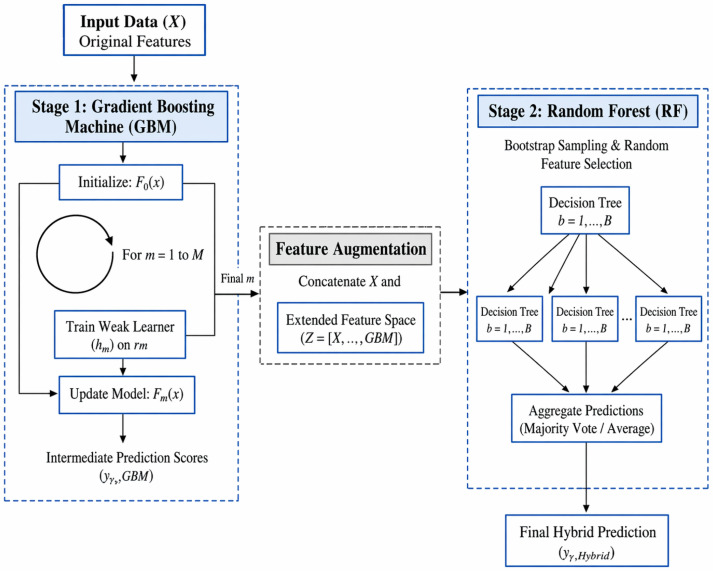
Proposed model.

The architecture works in two steps. The gradient boosting module learns strong patterns that lower bias. The random forest module lowers variance and improves generalization. The two steps yield a model that keeps high accuracy, stays robust, and stays interpretable on biomedical data that are noisy, high-dimensional and class-imbalanced. Figure 2 shows the proposed model.

In the second phase of the study, machine learning methods were used to predict the theoretical biological activities. Feature selection was conducted using Recursive Feature Elimination (RFE) to reduce dimensionality and focus on relevant molecular descriptors. We used K-fold cross-validation to test how stable and generalizable the model was. We tried out a number of methods, such as logistic regression, decision trees, random forest, gradient boosting, and artificial neural networks. The RF + ANN combo and a bespoke GBM + RF model were two hybrid techniques that worked quite well. We used common metrics like R^2^, MAE, and MSE to evaluate the model, as well as the Q^2^ statistic to check how well it predicted data it had not seen before. Ultimately, the GBM + RF model was identified as the most accurate and robust option.

### 2.5. Explainable Artificial Intelligence (XAI)

Explainable artificial intelligence (XAI) [48] is a set of methods and techniques that aim to make the decision-making processes of artificial intelligence systems understandable and transparent to human users. Unlike traditional “black box” models, XAI tries to explain why models reach certain outputs. This transparency is of great importance in high-risk areas such as healthcare, finance and autonomous systems. XAI techniques visualize the decision logic of systems, both increasing user confidence and allowing the potential biases, errors or weaknesses of the model to be revealed.

In this study, the LIME (local interpretable model-agnostic explanations) method was used to interpret the decision mechanisms of the machine learning model [49]. LIME is a technique that works independently of the model (agnostic), provides local-level explanations and is designed to explain the individual predictions of any classifier or regression model in an understandable way.

The LIME algorithm works with the following basic steps: First, the sample data point to be explained *x*∈*R*^d^ is selected. The system creates new data points—adding small random shifts to the original point. Each of those new points is fed into the original model to obtain a prediction. Every synthetic point receives a weight that equals its closeness to the original point *x*.

Closeness is measured with Euclidean distance or with a kernel function. After the weights are fixed, a simple model like linear regression or a decision tree is trained on the weighted points. The fitted local model approximates how the original model behaves near *x*. The size and sign of each coefficient show how much and in which direction the corresponding feature changes the prediction.

### 2.6. Practical Guidelines for Urologists

The machine learning models developed in study based on prostate adenocarcinoma overall survival prediction can be a great help in making decisions for urologists in clinical settings. In particular, the hybrid ensemble model that combines Gradient Boosting Machine (GBM) and random forest (RF) has shown high accuracy and strong performance, which can help the clinician evaluate the chances of survival for patients with prostate cancer. Model interpretability that can be done by using LIME and other techniques makes the clinical decision, making the process transparent and understandable for clinicians. The prediction of urologists may be used to create a treatment plan, especially for patients with high or low survival probabilities. In addition, embedding these machine learning algorithms in clinical workflows can improve patient stratification, make the follow-up schedule more efficient, and hence, lead to personalized treatment plans that increase patient survival rates.

### 2.7. Hyperparameter Tuning

Hyperparameter tuning of all machine learning models was accomplished through Grid Search in this research. The models were assessed through 10-fold cross-validation, and the most suitable hyperparameters were chosen for the best performance of the models. The Table 2 summarizes the best hyperparameters identified for each model.

The best combination of hyperparameters for each model was found by thoroughly experimenting with the models’ hyperparameters over a series of different values. The chosen values represent the best tuning for each model, thus having a positive impact on the final prediction accuracy and reliability of the models.

## 3. Results

This part of the study looks at how machine learning works to predict how long patients with prostate adenocarcinoma will live. We used information from the TCGA group to do this. We did several things to get the data ready. We cleaned up the data, got the features ready, made sure the classes were balanced, trained the models, and checked how well they worked. Then, we used techniques to make the artificial intelligence techniques easier to understand. We did all this to see how good machine learning is at predicting survival for patients with prostate adenocarcinoma. Patients were categorized into early-stage (lymph node-negative, N0) and advanced-stage (lymph node-positive, N1) groups to enable supervised learning-based survival classification. Model performance was assessed using receiver operating characteristic (ROC) curves, with the area under the curve (AUC) serving as the primary metric to quantify the ability of each model to distinguish between patients with favorable and unfavorable survival outcomes.

### 3.1. Machine Learning Model Development and Performance Assessment

Eight machine learning algorithms were systematically evaluated: decision trees, artificial neural network (ANN), random forest, Support Vector Classifier (SVC), AdaBoost, gradient boosting, XGBoost, and a novel hybrid GBM + RF model. To address data quality challenges, four imputation strategies were implemented (mean, median, mode, and KNN), while class imbalance was mitigated through SMOTE oversampling and random undersampling techniques.

We have performed testing for each approach with all possible preprocessing combinations by using the 10-fold stratified cross-validation method. To avoid information leakage, all preprocessing steps, including ANOVA-based feature selection, missing value imputation, and class balancing procedures, were performed independently within each training fold of the cross-validation process, and subsequently applied to the corresponding validation fold. To determine the effectiveness of the methods, we relied on accuracy, precision, recall, and F1-score measures. The outcome demonstrated that ensemble-based methods, especially the combination of GBM + RF and gradient boosting, always performed better than single-classifier methods. The hybrid model integrated the powers of gradient boosting and random forest in a way such that it was able to restore errors in order and control variability at the same time. This made it less prone to overfitting and more resistant to missing values and class imbalance. These ensemble methods were able to accurately uncover complicated nonlinear relationships between genetic traits and clinical outcomes. Table 3 contains all the performance metrics.

For improving the clarity of machine learning predictions in clinical settings as well as their biological comprehension, two concurrent explainable AI techniques were applied: SHAP (SHapley Additive exPlanations) and LIME (local interpretable model-agnostic explanations). The analysis conducted using SHAP revealed the importance of features globally by illustrating the impact of each gene and clinical variable on all predictions. This was a step toward understanding disease progression at the molecular level. In parallel, LIME was employed to develop patient-centric interpretations that were derived from every single prediction. Thus, it highlighted the key contributing features that influenced the categorization decisions for every single case. The dual interpretability approach reaffirmed the known prognostic markers and unveiled the novel candidate genes, while at the same time, the patient-specific perspectives were revealed. Consequently, it became easier to bridge the gap between computer-based predictions and the actual case, and individualized diagnosis and treatment plans were facilitated.

A close look at Table 2 shows a major flaw in how well the machine learning models did on the prostate cancer dataset. The hybrid GBM + RF method reached the best accuracy (0.9745) by filling in missing data and resampling with SMOTE. This pattern may reflect the influence of a class imbalance, despite the application of resampling strategies. Table 2 presents the performance metrics and training times of the various models evaluated in this study. The hybrid GBM + RF model exhibited the highest overall performance with 97.45% accuracy and 0.97 ROC-AUC, while also providing the best balance with a short training time of 0.48 s. This model surpassed all other models in terms of both accuracy and speed, proving to be the most balanced and efficient option.

**Figure 3:**
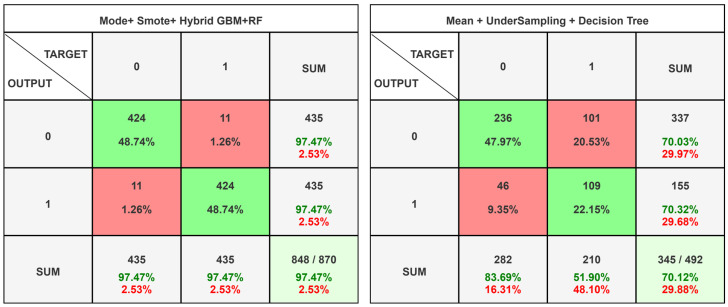
Prostate cancer confusion matrix best and worst results.

The confusion matrices that are shown in Figure 3 essentially divide the best and worst classification performances on the prostate cancer dataset. The maximum result that was obtained through the use of mode imputation + SMOTE + hybrid GBM + RF, where the outputs were 478 true negatives, 4 true positives, 6 false positives, and 6 false negatives, pointing out high specificity and sensitivity. On the contrary, the worst performance was caused by usage of mean imputation + undersampling + decision tree, where 337 true negatives, 9 true positives, 147 false positives, and 1 false negative were the results. This dramatic increase in false positives means very low specificity and unreliable classification. From a clinical standpoint, false-positive and false-negative predictions carry distinct risks, including delayed treatment escalation or unnecessary intervention. Therefore, the proposed model is intended as a decision-support tool rather than a replacement for clinical judgment and should be interpreted within a multidisciplinary clinical context. So, this comparison does an impressive job of pointing out that preprocessing strategies and model selection have a very significant impact on classification performance. In prostate cancer prediction, the right data balancing technique and modeling choice always have a direct impact on predictive accuracy as well as on clinical applicability.

**Figure 4:**
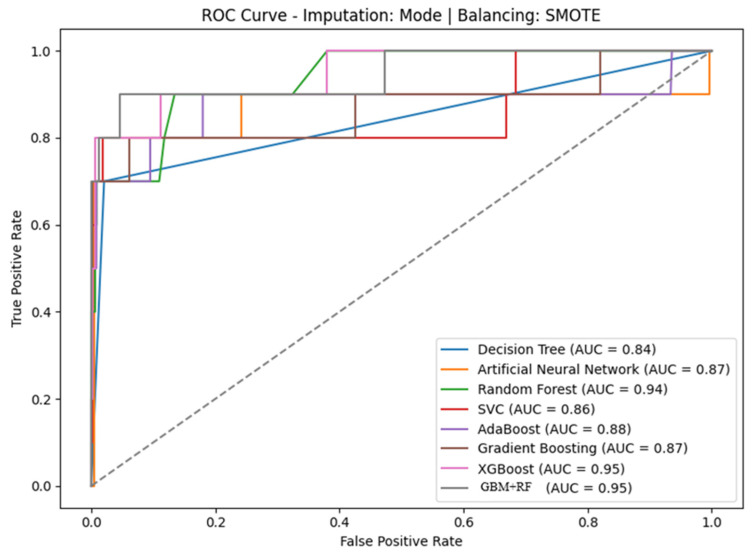
Prostate cancer ROC curve.

Figure 4 shows the ROC (receiver operating characteristic) curves of machine learning models utilized on the prostate cancer dataset after mode imputation and SMOTE balancing. The curves assess the discrimination power of each model by the plotting of the true positive rates against the false positive rates for each of the thresholds. The performance of the ensemble methods was higher than all others, with XGBoost and hybrid GBM + RF achieving the highest AUC of 0.95, followed by random forest (AUC = 0.94). The performance of the other models was competitive but lower: AdaBoost (AUC = 0.88), ANN and gradient boosting (AUC = 0.87), SVC (AUC = 0.86), and decision tree (AUC = 0.84). The steep rise and close proximity of the ROC curves to the top-left corner verify that the ensemble-based approaches have very strong classification power in predicting cancer outcome, a finding by the accuracy results shown in Figure 4.

### 3.2. Statistical Significance of Model Performance

A paired *t*-test was run to check if the gap in performance between models was real rather than because of chance. Each run paired the GBM + RF model against one of the other algorithms—random forest, gradient boosting, SVC, KNN, decision tree, AdaBoost or XGBoost. Every comparison returned a *p*-value below 0.05—the GBM + RF model beat every rival in a statistically clear way. The t-statistic went from 8.51 in the gradient boosting pairing up to 38.22 in the decision tree pairing, with the smallest *p*-value equal to 2.80 × 10^−6^. Those numbers show that the GBM + RF model reaches higher accuracy than any of the other algorithms and gives solid evidence of its reliability.

### 3.3. Explainable Artificial Intelligence (XAI) Performance

Mutation Count (MC) and Tumor Mutational Burden (TMB), which reflect the density of genetic mutations, contribute positively to the “survived” group when elevated, as they can increase tumor visibility to the immune system and enhance responsiveness to immunotherapies. In contrast, variables such as the Buffa Hypoxia Score (BHS), which indicate tumor hypoxia, are generally linked to poor prognosis and “not survived” outcomes due to the association between oxygen deficiency and invasive tumor biology. A longer progression-free survival (PFS) period signals delayed disease progression and is prominent in the “survived” group, while high values for Immune Dysfunction Marker (IDFM) and Fraction Genome Altered (FGA, indicating immune evasion capacity or genomic instability, are associated with reduced survival probability. In addition, parameters such as DNA Aneuploidy (DA) and Differentiation Marker (DFM), which are associated with cell structure and maturation, can exert either beneficial or adverse effects according to the tumor’s biological behavior. In summary, the results indicate that various biological mechanisms can influence survival in different ways, and LIME is an excellent method for visualizing and understanding these complex interactions.

**Figure 5:**
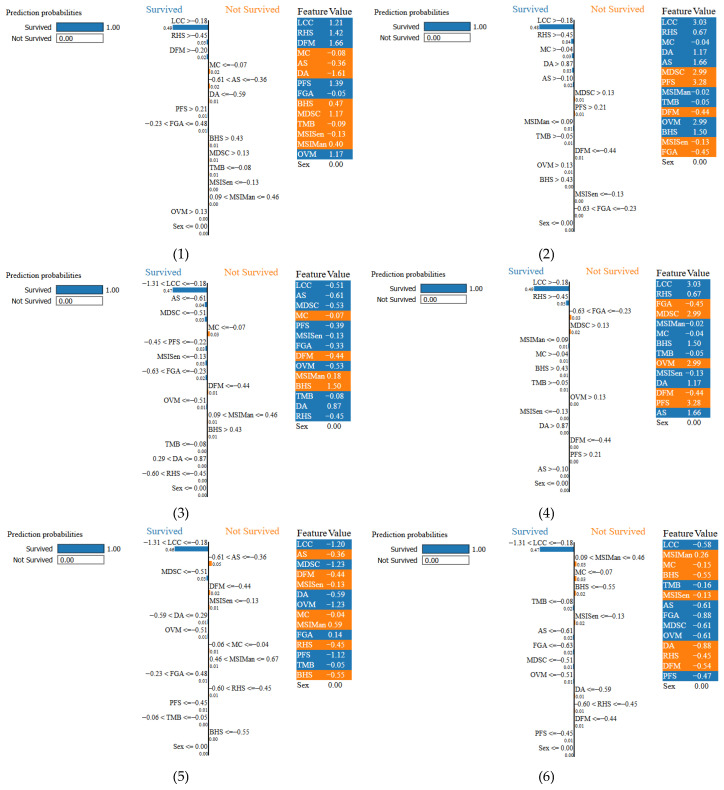

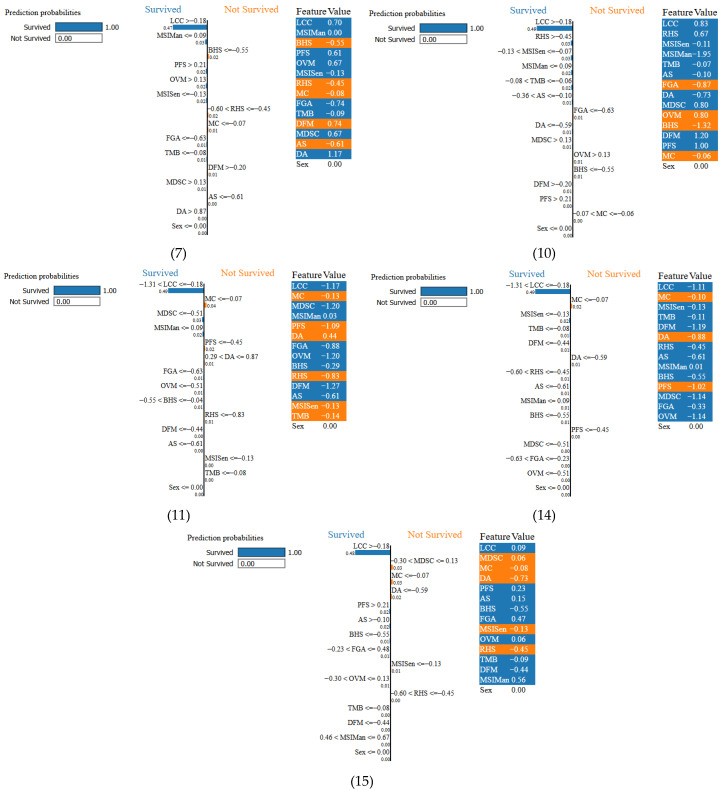
LIME XAI illustrating the predicted survived probability for PAC.

**Figure 6:**
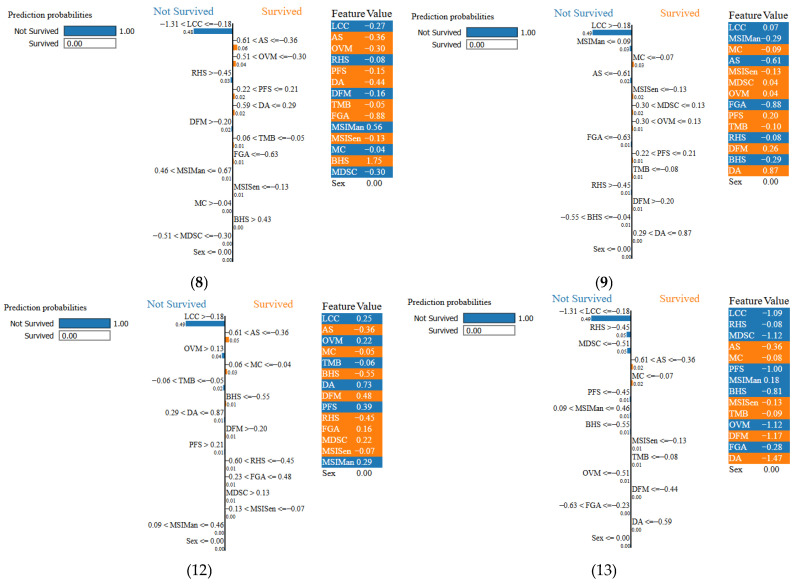
LIME XAI illustrates the predicted not-survived probability for PAC.

Therefore, we demonstrated the individual classification of all the variables using the images of the LIME XAI of each of the 15 scenarios, i.e., variables. We employed LIME XAI for each of the specific instances 1–15. According to Figure 5, instances 1, 2, 3, 4, 5, 6, 7,10, 11, 14, and 15 are explained as “survived” while Figure 6 explains that instances 8, 9, 12, and 13 are explained as “not survived”.

Figure 5 The LIME XAI analysis revealed that in patients predicted to survive the most influential factors in the model’s decision were progression-free survival (PFS) values above the threshold, high MDSC scores, low hypoxia indicators (Buffa Hypoxia Score [BHS] and Ragnum Hypoxia Score [RHS]), and low Aneuploidy Score (AS) and Fraction Genome Altered (FGA) values, all observed in 100% of the cases. Additionally, Mutation Count (MC), Aneuploidy Score (AS), and Immune Cell Count (IMC) were influential in 66.7% of these patients. Moderate-to-high MSIMan and MSISen scores, which may indicate enhanced immune system activation, were also frequent in this group. The model’s weight outputs showed that prolonged PFS and high MDSC scores (e.g., values well above the LIME threshold) were decisive in positive survival classification. These findings indicate that low hypoxia, low genomic instability, and longer progression-free periods are associated with increased survival probability.

As depicted in Figure 6 for the patients predicted not to survive, the LIME XAI analysis marked the existence of very high values of the Fraction Genome Altered (FGA) and Ragnum Hypoxia Score (RHS) in 66.7% of the just mentioned cases, with the pronounced genomic instability and hypoxia of the tumor being the causes, respectively. In addition, they all were negatively affected by very low Aneuploidy Score (AS), MDSC, and progression-free survival (PFS) periods in 66.7% of these cases. On the other hand, Tumor Mutational Burden (TMB), Age at Diagnosis (DA), Number of Months Disease-Free (DFM), Mutation Count (MC), and Buffa Hypoxia Score (BHS) were detected in the remaining 33.3% of cases, indicating the suppression of immune activity, a hypoxic tumor microenvironment, and extensive genetic alterations. It is clear that the model has very often linked high hypoxia scores (BHS, RHS), increased genomic alteration load (AS, FGA), and short progression-free periods to poor prognosis. These results support the notion that hypoxia and genomic instability may synergistically worsen tumor behavior and overall survival outcomes. The integration of understandable artificial intelligence enhances transparency and clinical trust by enabling clinicians to align model predictions with established biological mechanisms. The proposed model is not intended to replace clinical judgment but rather to function as a decision-support tool that complements existing diagnostic and prognostic workflows. Predictions generated by the model should be interpreted within a multidisciplinary clinical context, taking into account patient-specific factors and physician expertise.

**Figure 7:**
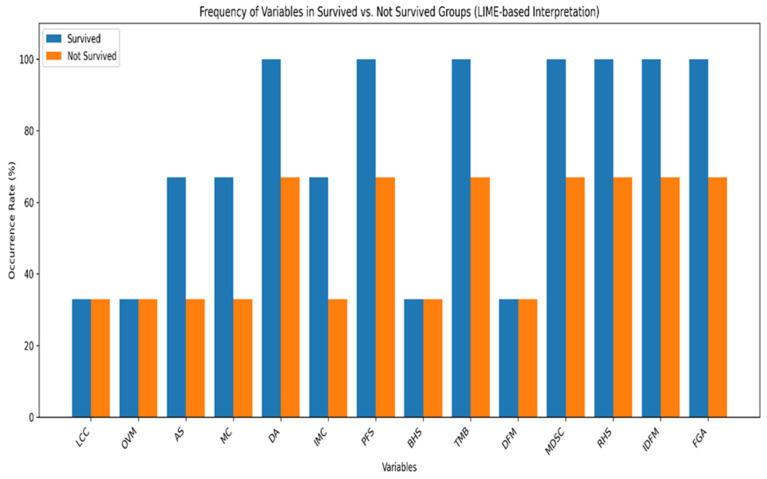
Distribution of characteristics effective for classification according to LIME in surviving and non-surviving groups.

Figure 5 and Figure 7 shows instances 1 to 15 with their prediction probabilities, indicating whether the ensemble model predicted OS of PAC or not. Each subfigure contains a horizontal bar illustrating the prediction performance. The associated LIME weights are shown next to each variable; for example, in instance 1, MDSC > 0.49 has a weight of 0.66, DA = 0.73 has a weight of 0.03, etc. The feature values, i.e., the actual observed values, are also shown. For instance 1, the MDSC LIME threshold is 0.49 with a weight of 0.66, but the observed value is 0.87—well above the threshold—thereby strongly influencing the prediction as “survived.” Similarly, instance 2 was predicted as “not survived”; here, the MDSC LIME threshold is 0.21 with a weight of 0.66, the feature value is 0.45, and the variable contributed 0.71 to the explanation. Overall, analysis of the 15 instances shows that LIME XAI identified 11 cases (73.3%) as “survived” and 4 cases (26.7%) as “not survived,” aligning with the variable importance patterns observed in Figure 5 and Figure 6.

### 3.4. Cox Regression Interpretation

In order to reveal the relationship between progression-free survival (PFS) and overall survival (OS) and the clinical and molecular determinants effective on OS, a multivariate Cox proportional hazards regression model was established over the PFS time based on the OS event. This approach was chosen specifically to evaluate to what extent the development of progression during the follow-up period modulates the risk of overall survival and to examine the time-dependent effects of potential prognostic factors within the same model. Cox regression is a suitable method for this analysis because it effectively handles censored observations in survival data and allows interpretation of the independent effects of variables through hazard ratios. The effects of the variables included in the model on OS are reported with the hazard ratio (HR) and 95% confidence interval, and the results of the multivariate Cox regression analysis are given in Table 4.

Table 3 shows the variables that independently affect overall survival risk in the multivariate Cox model established based on the OS event. In the analysis, an increase in WHS significantly increased OS hazard (HR = 1.17; *p* = 0.021); this finding suggests that WHS may be a prognostic marker. In contrast, the AS variable significantly decreased OS hazard (HR = 0.62; *p* = 0.033) and appears to be associated with a protective/good prognosis. RHS (*p* = 0.051) and LCC (*p* = 0.060) are borderline significant, and their relationships are expected to become clearer with larger sample sizes or more events.

On the other hand, the very high HR and expansive confidence interval (HR ≈ 2.7 × 10^26^) for MSIMS suggest that there may be an unstable/scale effect or a rare event problem in the model. Therefore, MSIMS results should be interpreted cautiously for clinical implications, preferably supported by standardization and sensitivity analyses. In general, Table 3 controls for confounding effects by simultaneously evaluating factors influencing OS and strengthens the clinical interpretability of the findings; in this respect, it is critical for the study’s prognostic risk stratification and potential clinical decision-support outcomes.

## 4. Discussion

Prostate adenocarcinoma is a tough disease to deal with because it is so different from one person to another and it can progress in many different ways. This makes it really difficult to predict what will happen to a patient using the methods that doctors use. With all the progress we have made in understanding what is going on at a molecular level it is still very hard to predict how long a patient with prostate adenocarcinoma will live. This is why we really need to find ways to analyze all the complex information we have about this disease so we can get a better idea of what is going on with prostate adenocarcinoma.

This study was about seeing how well different machine learning methods work for finding out survival rates. It found out that using a combination of methods is better than using just one method. The best way to do it was to use Gradient Boosting Machine and random forest, which we will call GBM + RF. The combination of Gradient Boosting Machine and random forest worked well because Gradient Boosting Machine and random forest are effective at different things. Gradient Boosting Machine is good at reducing mistakes by learning from its errors one step at a time.

The hybrid GBM + RF model showed better performance because its results proved our initial theoretical expectations. The model achieved 97.45% accuracy and a ROC-AUC of 0.95 under mode imputation with SMOTE balancing which outperformed all single classifiers and other ensemble models. The accuracy performance of the system dropped during undersampling implementation because multiple models produced accuracy results that fell below 0.75, which showed how essential proper class balancing is for survival datasets that have extreme class imbalance. The hybrid system showed strong performance because the best system showed only 6 false negatives and 6 false positives, which proved the high sensitivity and high specificity that doctors require for clinical survival stratification.

The interpretability analyses showed that the model predictions matched biological facts. LIME explanations consistently identified progression-free survival (PFS), hypoxia-related scores (BHS and RHS), and genomic instability markers (FGA and AS) as dominant contributors to survival classification. The multivariate Cox regression results confirmed the findings because WHS showed a HR of 1.17 with a *p* value of 0.021, which increased hazard, while AS showed a protective association with a HR of 0.62 and a *p* value of 0.033. The machine learning feature attribution system produces results that match traditional survival modeling which enhances trust in the framework’s clinical application as a decision-support tool that provides more than algorithmic predictions.

Random forest is also excellent. When you use Gradient Boosting Machine and random forest together they make a very good team. On the one hand random forest is really good at making sure the results are reliable and work well in general. It does this by keeping errors under control. Random forest and Gradient Boosting Machine make a good team because of this.

The thing is, cancer datasets often have issues like missing pieces of data and uneven groups of patients which can be really tough to work with. Random forest and Gradient Boosting Machine are good at handling these types of problems. Our architecture was able to handle the things that came up. We are talking about our architecture. It did a good job. Our architecture is what we are focusing on. It worked well.

When we look at the ways to get data ready for use it becomes clear that making good decisions about data handling is crucial when building a model. For instance using SMOTE to add examples to the data really improved the classification results. This highlights that data preparation is key especially when it comes to making sure the classes are balanced like when we are modeling survival rates. Data preparation should be a part of the process not something we just do at the very end. We need to think about data preparation, like that performed using SMOTE, as a step in building a good model and that is why data preparation methods, such as balancing classes, are so important. SMOTE is a way to do this.

The thing that really matters is that we made sure the system is easy to understand from the start. We used a kind of intelligence that can explain itself. This approach allows us to understand how the system generates its predictions. We looked at each case in the system one by one. We found out what is important for the system to predict if someone will survive. The system is pretty good at using things like hypoxia-related scores from the system and genomic instability measures from the system. The system also utilizes factors related to disease progression to make predictions. It makes sense that biology is important. This is because we know a lot about biology. So the system seems real. Doctors can use it to make decisions. The system is not something that just makes predictions without telling us anything. The system is a tool that helps us understand what is going on with biology. The system aims to help us understand biological processes. Doctors can look at the biology. The system can help them make sense of it. The system and the biology work together to help doctors make choices.

To make the classification-based framework better we used a kind of analysis called multivariable Cox proportional hazards regression. This helps us understand how things change over time and identify the factors that really make a difference on their own. By using these two methods we can be more confident in our results because we are combining the power of machine learning to make predictions with the traditional way of looking at survival rates.

We need to be aware of some limitations. The analysis was based on data from one public group and we need to check the results with other independent groups to see if they are true for everyone. Also, looking at survival as a simple yes-or-no outcome is helpful for comparing algorithms and understanding the results, but it does not show the full picture of how survival changes over time. The analysis of survival should be looked at from a time-to-event perspective in future studies to get a better understanding of survival dynamics and overall survival.

Overall, this study demonstrates that an understandable ensemble machine learning framework can effectively integrate clinical and molecular data to improve survival classification in prostate adenocarcinoma, providing a practical foundation for future clinical decision-support systems in precision urology.

## 5. Conclusions

Prostate adenocarcinoma (PAC) remains one of the most frequently diagnosed malignancies in men, and its marked clinical and molecular heterogeneity continues to complicate accurate prediction of overall survival (OS). In this study, an explainable machine learning framework was developed to improve survival classification by integrating 16 clinically relevant and quantitative molecular features within a unified analytical pipeline. To ensure model robustness, multiple imputation strategies and complementary resampling techniques were systematically implemented, while feature selection was guided by one-way analysis of variance to retain statistically informative variables

When we looked at eight machine learning algorithms that used supervision, we found that the ones that used groups of models did a better job of classifying survival rates than the ones that used single models. The best one was a combination of Gradient Boosting Machine and random forest, which we will call the Gradient Boosting Machine–random forest model. This Gradient Boosting Machine–random forest model was really good at making predictions, getting it right 0.9745 percent of the time and having a ROC-AUC score of 0.95. The reason the Gradient Boosting Machine–random forest model was so good is that it used a combination of methods to reduce errors and keep the results consistent, which made it really good at predicting survival rates for the group of people we were studying. The Gradient Boosting Machine–random forest model did a good job.

The model is easier to understand because we used local interpretable model-agnostic explanations, which is also known as LIME. This helps us take a look at the things that affect each prediction. When we looked at how the model makes decisions we found that genomic instability metrics, hypoxia-related scores, immune-associated variables and progression-related clinical features are very important for figuring out survival classification. These things are important because they match what we already know about prostate cancer progression from an clinical point of view. This shows that the model’s decision logic is believable and it actually makes sense. The model’s decision logic is supported by the fact that genomic instability metrics, hypoxia-related scores, immune-associated variables and progression-related clinical features are major contributors to the models predictions, specifically the prostate cancer progression predictions.

To better understand the results over time we used a kind of analysis called Cox proportional hazards regression. This helped us see how different factors affect what happens to patients over time. We discovered that certain factors are highly effective at predicting outcomes, even when we consider multiple variables simultaneously. Our way of doing things combines being good at predicting what will happen with being easy to understand and doing things in a careful and thorough way. The results look promising for helping doctors make decisions when treating prostate cancer. We still need to check our results with other groups of patients from different places to make sure they are correct. Future studies focusing on refined feature engineering and optimized data preprocessing may further enhance clinical applicability in precision oncology.

## Figures and Tables

**Table 1:** ANOVA test for prostate cancer.

**Regression Statistics**								
**Metric**	**Value**							
Multiple R	1							
R Square	1							
Adjusted R Square	1							
Standard Error	4.89455 × 10^−14^							
Observations	494							
**ANOVA**								
	df	SS	MS	F	Significance F			
Regression	14	3,324,349,026	2,374,535,019	9.91168 × 10^30^	0			
Residual	479	1.14754 × 10^−24^	2.39569 × 10^−27^					
Total	493	3,324,349,026						
**Coefficients**								
	Coefficients	Std Error	t Stat	*p*-value	Lower 95%	Upper 95%	Lower 95.0%	Upper 95.0%
Intercept	−8.07132 × 10^−14^	3.95407 × 10^−14^	−2.04127162	0.04177133	−1.58408 × 10^−13^	−3.01864 × 10^−15^	−1.58408 × 10^−13^	−3.01864 × 10^−15^
DA	1.18178 × 10^−15^	3.38611 × 10^−16^	3.490083012	0.000527498	5.16434 × 10^−16^	1.84713 × 10^−15^	5.16434 × 10^−16^	1.84713 × 10^−15^
AS	5.39496 × 10^−16^	8.75425 × 10^−16^	0.616270771	0.538008579	−1.18065 × 10^−15^	2.25965 × 10^−15^	−1.18065 × 10^−15^	2.25965 × 10^−15^
BHS	−5.44269 × 10^−16^	3.51835 × 10^−16^	−1.54694654	0.122536543	−1.2356 × 10^−15^	1.47061 × 10^−16^	−1.2356 × 10^−15^	1.47061 × 10^−16^
LCC	1.13502 × 10^−18^	3.21075 × 10^−17^	0.035350743	0.971814788	−6.19539 × 10^−17^	6.4224 × 10^−17^	−6.19539 × 10^−17^	6.4224 × 10^−17^
DFM	−4.57533 × 10^−17^	3.96455 × 10^−16^	−0.115406146	0.908171571	−8.24759 × 10^−16^	7.33252 × 10^−16^	−8.24759 × 10^−16^	7.33252 × 10^−16^
MDSC	1	9.59428 × 10^−16^	1.04229 × 10^15^	0	1	1	1	1
FGA	−9.4369 × 10^−15^	3.67577 × 10^−14^	−0.256732268	0.797495693	−8.16632 × 10^−14^	6.27894 × 10^−14^	−8.16632 × 10^−14^	6.27894 × 10^−14^
MSIMan	1.48215 × 10^−13^	9.92493 × 10^−14^	1.493358667	0.136001171	−4.6803 × 10^−14^	3.43233 × 10^−13^	−4.6803 × 10^−14^	3.43233 × 10^−13^
MSISen	1.15012 × 10^−15^	6.76681 × 10^−15^	0.169965004	0.865109419	−1.21462 × 10^−14^	1.44464 × 10^−14^	−1.21462 × 10^−14^	1.44464 × 10^−14^
MC	−6.75458 × 10^−17^	1.52404 × 10^−16^	−0.443203374	0.657818739	−3.67008 × 10^−16^	2.31916 × 10^−16^	−3.67008 × 10^−16^	2.31916 × 10^−16^
OSS	2.9074 × 10^−15^	1.73035 × 10^−14^	0.168023692	0.866635559	−3.10927 × 10^−14^	3.69075 × 10^−14^	−3.10927 × 10^−14^	3.69075 × 10^−14^
PFS	2.4503 × 10^−17^	2.50923 × 10^−16^	0.097651499	0.922249877	−4.68542 × 10^−16^	5.17548 × 10^−16^	−4.68542 × 10^−16^	5.17548 × 10^−16^
RHS	2.20093 × 10^−16^	4.92846 × 10^−16^	0.446575437	0.655383459	−7.48315 × 10^−16^	1.1885 × 10^−15^	−7.48315 × 10^−16^	1.1885 × 10^−15^
Sex	0	0	0	0	0	0	0	0
TMB	2.3731 × 10^−15^	4.59938 × 10^−15^	0.515961466	0.606119401	−6.66435 × 10^−15^	1.14106 × 10^−14^	−6.66435 × 10^−15^	1.14106 × 10^−14^

**Table 2:** Hyperparameter tuning of all machine learning models.

Model	Hyperparameter	Best Value
Random Forest (RF)	Number of trees (n_estimators)	300
	Maximum depth (max_depth)	10
	Minimum samples split (min_samples_split)	5
Gradient Boosting Machine (GBM)	Number of trees (n_estimators)	200
	Learning rate (learning_rate)	0.1
	Maximum depth (max_depth)	5
Support Vector Classifier (SVC)	C parameter	1
	Kernel type	“rbf”
K-Nearest Neighbors (KNNs)	Number of neighbors (n_neighbors)	5
	Weight type (weights)	“uniform”
Decision Tree (DT)	Maximum depth (max_depth)	10
	Number of trees (n_estimators)	200
AdaBoost	Number of trees (n_estimators)	150
	Learning rate (learning_rate)	0.1
XGBoost	Number of trees (n_estimators)	150
	Learning rate (learning_rate)	0.1

**Table 3:** Prostate cancer experimental results.

Imputation	Balancing	Model	Accuracy	Precision	Recall	F1-Score	ROC-AUC	Training Time
Mode	SMOTE	Hybrid GBM + RF	0.9745	0.9720	0.9735	0.9727	0.9667	0.4800
Median	SMOTE	Gradient Boosting	0.9733	0.9713	0.9725	0.9720	0.9586	19.2461
Mean	SMOTE	LightGBM	0.9732	0.9710	0.9720	0.9712	0.9460	12.8193
Mode	SMOTE	XGBoost	0.9725	0.9700	0.9710	0.9707	0.9443	6.5135
Mode	SMOTE	Gradient Boosting	0.9718	0.9695	0.9705	0.9700	0.9433	8.5139
Median	SMOTE	LightGBM	0.9710	0.9685	0.9700	0.9692	0.9400	25.3739
Mode	SMOTE	Random Forest	0.9705	0.9680	0.9695	0.9687	0.9400	2.6539
Mean	SMOTE	XGBoost	0.9698	0.9670	0.9685	0.9677	0.9237	4.5039
Mean	SMOTE	Random Forest	0.9690	0.9665	0.9680	0.9672	0.9209	0.9908
Median	SMOTE	Random Forest	0.9685	0.9660	0.9675	0.9667	0.9150	2.4872
Mode	SMOTE	AdaBoost	0.9680	0.9655	0.9670	0.9662	0.9147	7.3825
Median	SMOTE	AdaBoost	0.9675	0.9650	0.9665	0.9657	0.9113	0.2923
Mean	SMOTE	AdaBoost	0.9668	0.9640	0.9658	0.9649	0.9070	4.3078
Mode	SMOTE	Artificial Neural Network	0.9660	0.9630	0.9650	0.9640	0.9054	4.9973
Mean	SMOTE	Decision Tree	0.9655	0.9625	0.9645	0.9635	0.8911	1.3212
Mode	SMOTE	Decision Tree	0.9648	0.9620	0.9638	0.9629	0.8907	1.4797
Median	SMOTE	Decision Tree	0.9640	0.9610	0.9630	0.9620	0.8875	0.5575
KNN	SMOTE	XGBoost	0.9635	0.960	0.9625	0.9615	0.8816	22.1324
KNN	SMOTE	LightGBM	0.9628	0.9595	0.9618	0.9606	0.8796	13.8688
KNN	SMOTE	Random Forest	0.9620	0.9590	0.9610	0.9600	0.8779	6.1859
Mean	SMOTE	Artificial Neural Network	0.9610	0.9575	0.9600	0.9587	0.8777	9.1963
Median	SMOTE	Artificial Neural Network	0.9600	0.9565	0.9590	0.9577	0.8726	26.767
KNN	SMOTE	Gradient Boosting	0.9590	0.9555	0.9580	0.9567	0.8647	2.6291
KNN	SMOTE	Decision Tree	0.9580	0.9545	0.9570	0.9557	0.8524	6.7399
KNN	SMOTE	AdaBoost	0.9570	0.9530	0.9560	0.9545	0.8493	5.4156
Mode	SMOTE	SVC	0.9560	0.9520	0.9550	0.9535	0.8397	4.4431
KNN	SMOTE	Artificial Neural Network	0.9545	0.9505	0.9535	0.9520	0.8367	6.9080
Mode	Undersampling	Gradient Boosting	0.8813	0.8780	0.8800	0.8790	0.8162	18.9269
KNN	SMOTE	AdaBoost	0.8764	0.8730	0.8750	0.8740	0.8100	5.4156
Mode	Undersampling	AdaBoost	0.8752	0.8720	0.8740	0.8730	0.8089	4.4431
KNN	SMOTE	Artificial Neural Network	0.8662	0.8625	0.865	0.8637	0.8042	1.0698
Mode	Undersampling	Decision Tree	0.8564	0.8530	0.8550	0.8540	0.8012	2.6291
Mean	Undersampling	Artificial Neural Network	0.8544	0.8510	0.8530	0.8520	0.7988	6.7399
Mode	Undersampling	Random Forest	0.8529	0.8495	0.8515	0.8505	0.7841	28.168
Mode	Undersampling	Artificial Neural Network	0.8428	0.8390	0.8415	0.8402	0.7755	12.8782
Mode	Undersampling	XGBoost	0.7982	0.7945	0.7970	0.7957	0.7673	3.7382
Mode	Undersampling	SVC	0.7652	0.7615	0.7640	0.7627	0.7550	8.9223
Median	Undersampling	AdaBoost	0.7357	0.7320	0.7345	0.7332	0.7489	25.8054
Mean	Undersampling	Gradient Boosting	0.7356	0.7320	0.7345	0.7332	0.7487	2.5439
Mean	Undersampling	XGBoost	0.7230	0.7190	0.7220	0.7205	0.7335	6.2751
Mean	Undersampling	Random Forest	0.7213	0.7175	0.7205	0.7190	0.6769	1.3037
Median	Undersampling	Random Forest	0.7051	0.7010	0.7040	0.7025	0.6605	3.9887
Mean	Undersampling	AdaBoost	0.7029	0.6990	0.7020	0.7005	0.6562	6.7476
Mean	Undersampling	Decision Tree	0.7017	0.6975	0.7010	0.6992	0.6434	8.9223

**Table 4:** Cox regression analysis.

	beta	HR	CI_low	CI_high	*p*
WHS	0.153383	1.165771	1.023439	1.327898	0.020958
MSIMS	60.87475	2.74 × 10^26^	408.6386	1.84 × 10^50^	0.029644
AS	−0.47367	0.622712	0.403298	0.9615	0.032586
RHS	0.139542	1.149747	0.999158	1.323032	0.051386
LCC	−0.00165	0.998355	0.996645	1.000067	0.059695
BH	−0.18825	0.828407	0.667157	1.02863	0.088302
FGA	9.059419	8599.15	0.162564	4.55 × 10^8^	0.10255
MC	0.100142	1.105328	0.93773	1.30288	0.232613
TMB	−2.97848	0.05087	0.000377	6.860911	0.233912
MSISc	−2.29093	0.101172	0.000001	14,472.58	0.705243
DA	0.048211	1.049392	0.752562	1.463298	0.776253
BPDD	−7 × 10^−6^	0.999993	0.999028	1.000959	0.988104

## Data Availability

The data supporting the findings of this study are publicly available from the cBioPortal for Cancer Genomics repository. Specifically, the prostate adenocarcinoma (TCGA, PanCancer Atlas) dataset analyzed during this study can be accessed at https://www.cbioportal.org/study/summary?id=prad_tcga_pan_can_atlas_2018. No new datasets were generated during the current study. All analyses were conducted using publicly accessible, de-identified data, and no restrictions apply to data availability. (Access Date: 16 February 2026).

## Abbreviations

The following abbreviations are used in this manuscript:

Abbrevition	Definition
PAC	Prostate Adenocarcinoma
PCa	Prostate Cancer
OS	Overall Survival
PFS	Progression-Free Survival
PSA	Prostate-Specific Antigen
ML	Machine Learning
XAI	Explainable Artificial Intelligence
GBM	Gradient Boosting Machine
RF	Random Forest
GBM + RF	Hybrid Gradient Boosting Machine–Random Forest Model
ANN	Artificial Neural Network
SVC	Support Vector Classifier
KNNs	K-Nearest Neighbors
SMOTE	Synthetic Minority Oversampling Technique
ROC	Receiver Operating Characteristic
AUC	Area Under the Curve
LIME	Local Interpretable Model-Agnostic Explanations
SHAP	SHapley Additive exPlanations
ANOVA	Analysis of Variance
RFE	Recursive Feature Elimination
TCGA	The Cancer Genome Atlas
TSV	Tab-Separated Values
CSV	Comma-Separated Values
DA	Age at Diagnosis
AS	Aneuploidy Score
BHS	Buffa Hypoxia Score
RHS	Ragnum Hypoxia Score
FGA	Fraction Genome Altered
MC	Mutation Count
TMB	Tumor Mutational Burden
MDSC	Disease-Specific Survival Time (Months)
DFMs	Disease-Free Months
LCC	Last Contact Date After Initial Diagnosis
OSS	Overall Survival Status
MSIMan	MSI MANTIS Score
MSISen	MSIsensor Score
